# Vaccine effectiveness against hospitalisation estimated using a test-negative case-control study design, and comparative odds of hospital admission and severe outcomes with COVID-19 sub-lineages BQ.1, CH.1.1. and XBB.1.5 in England

**DOI:** 10.1016/j.lanepe.2023.100755

**Published:** 2023-10-26

**Authors:** Freja Cordelia Møller Kirsebom, Katie Harman, Rachel Jayne Lunt, Nick Andrews, Natalie Groves, Nurin Abdul Aziz, Russell Hope, Julia Stowe, Meera Chand, Mary Ramsay, Gavin Dabrera, Meaghan Kall, Jamie Lopez Bernal

**Affiliations:** a UK Health Security Agency, London, United Kingdom; b NIHR Health Protection Research Unit in Vaccines and Immunisation, London School of Hygiene and Tropical Medicine, London, United Kingdom; c Guys and St Thomas’s Hospital NHS Trust, London, United Kingdom; d NIHR Health Protection Research Unit in Respiratory Infections, Imperial College London, London, United Kingdom

**Keywords:** COVID-19, Vaccine effectiveness, Test-negative case-control, Variant severity, Omicron

## Abstract

**Background:**

Since the first emergence of Omicron BA.1 in England in November 2021, numerous sub-lineages have evolved. In September 2022, BA.5 dominated. The prevalence of BQ.1 increased from October, while the prevalence of CH.1.1 and XBB.1.5 increased from December 2022 and January 2023, respectively. Little is known about the effectiveness of the vaccines against hospitalisation with these sub-lineages, nor the relative severity, so we here used national-level electronic health records from England to estimate vaccine effectiveness and variant severity.

**Methods:**

The study period for tests contributing to all analyses was from 5th December 2022 to 2nd April 2023, when the variants of interest were co-circulating. A test-negative case-control study was used to estimate the incremental effectiveness of the bivalent BA.1 booster vaccines against hospitalisation, relative to those with waned immunity where the last dose was at least 6 months prior. The odds of hospital admission for those testing PCR positive on the day of an attendance to accident and emergency departments and the odds of intensive care unit admission or death amongst COVID-19 admissions were compared between variants. Additionally, a Cox proportional hazards survival regression was used to investigate length of stay amongst hospitalised cases by variant.

**Findings:**

Our vaccine effectiveness study included 191,229 eligible tests with 1647 BQ.1 cases, 877 CH.1.1 cases, 1357 XBB.1.5 cases and 187,348 test negative controls. There was no difference in incremental vaccine effectiveness against hospitalisation with BQ.1, CH.1.1 or XBB.1.5, nor was there a difference in the severity of these variants. Effectiveness against hospitalisation was 48.0% (95% C.I.; 38.5–56.0%), 29.7% (95% C.I.; 7.5–46.6%) and 52.7% (95% C.I.; 24.6–70.4%) against BQ.1, CH.1.1 and XBB.1.5, respectively, at 5–9 weeks post booster vaccination. Compared to BQ.1, the odds of hospital admission were 0.87 (95% C.I.; 0.77–0.99) and 0.88 (95% C.I.; 0.75–1.02) for CH.1.1 and XBB.1.5 cases attending accident and emergency departments, respectively. There was no significant difference in the odds of admission to intensive care units or death for those with CH.1.1 (OR 0.96, 95% C.I.; 0.71–1.30) or XBB.1.5 (OR 0.67, 95% C.I.; 0.44–1.02) compared to BQ.1. There was also no significant difference in the length of hospital stay by variant.

**Interpretation:**

Together, these results provide reassuring evidence that the bivalent BA.1 booster vaccines provide similar protection against hospitalisation with BQ.1, CH.1.1 and XBB.1.5, and that the emergent CH.1.1 and XBB.1.5 sub-lineages do not cause more severe disease than BQ.1.

**Funding:**

None.

**Research in context**

**Evidence before this study**

The Omicron variant of SARS-Cov-2 has evolved into distinct sub-lineages which have caused numerous infection waves in the UK. Most recently, the prevalence of BQ.1/BQ.1.1 increased from October 2022, while the prevalence of CH.1.1 and XBB.1.5 increased from December 2022 and January 2023, respectively. As part of the UK COVID-19 vaccination programme, bivalent BA.1 boosters were offered from 5th September 2022 to all adults aged 50 years and over and vulnerable individuals including the immunosuppressed. We searched PubMed using the terms ‘COVID-19’, ‘bivalent’, ‘vaccine’, ‘vaccine effectiveness’, ‘variant’ and ‘severity’ with no date restrictions in July 2023 and used the snowball process to identify additional relevant publications. We also scoped preprint databases (MedXriv) for relevant COVID-19 vaccine effectiveness studies and variant severity studies undertaken during the autumn/winter of 2022/2023. Both CH.1.1 and XBB.1.5 have demonstrated growth advantages and proven to be highly transmissible but there is limited evidence available on the severity of CH.1.1 and XBB.1.5. To our knowledge, there are no real-world estimates of VE against BQ.1 or CH.1.1. Studies from the United States found VE against XBB/XBB.1.5 was generally comparable to that seen against BA.5, while a study from Singapore found protection against A&E attendance was 49% during an XBB wave.

**Added value of this study**

We here use national-level electronic healthcare records to assess vaccine effectiveness and variant severity in England between 5th December 2022 to 2nd April 2023, when the variants of interest were co-circulating. We found no difference in the protection conferred by the bivalent BA.1 booster vaccines against hospitalisation with BQ.1, CH.1.1 and XBB.1.5. Point estimates for odds of severe disease indicators with CH.1.1 and XBB.1.5 were generally lower than for BQ.1, both for the odds of hospital admission or death following A&E attendance and for odds of ICU admission or death among hospitalised patients, though in most analyses this was not statistically significant. The length of stay following hospital admission also did not differ by variant.

**Implications of all the available evidence**

Together, these results provide reassuring evidence that the bivalent BA.1 booster vaccines provide similar protection against hospitalisation with BQ.1, CH.1.1 and XBB.1.5, and that the emergent CH.1.1 and XBB.1.5 sub-lineages do not cause more severe disease than BQ.1. The analyses follow previously observed trends showing similarity in the vaccine effectiveness against, and severity of, Omicron sub-lineages.

## Introduction

The first Omicron sub-lineage to emerge in the UK was BA.1 in November 2021,^1^ followed by BA.2^2^ and BA.4 and BA.5.^3^ In the autumn/winter of 2022/23, BA.5 dominated in September. The prevalence of BQ.1/BQ.1.1 increased from October,^4^ while the prevalence of CH.1.1 and XBB.1.5 increased from December 2022 and January 2023, respectively^5^ (Supplementary Fig. S1). These sub-lineages have all acquired different combinations of mutations in the spike protein as compared to BA.1.^6^^,^^7^ Both CH.1.1 and XBB.1.5 have demonstrated growth advantages and proven to be highly transmissible sub-lineages of Omicron.^7^

Previous Omicron sub-lineages have shown no increase in severity, including BA.4 and BA.5 compared to BA.2, and BA.4.6, BA.2.75 and BQ.1 compared to BA.5,^8^^,^^9^ but there is limited evidence available on the severity of CH.1.1 and XBB.1.5. Initial data suggested XBB.1.5 has a similar level of severity compared to the baseline of BQ.1.^10^ Evidence from laboratory-based assessments of the efficacy of therapeutic monoclonal antibodies against BQ.1, BA.2.75.2 (parental lineage of CH.1.1) and XBB (parental lineage of XBB.1.5), as well as studies evaluating the neutralising ability of plasma antibodies from vaccinated individuals against these variants have suggested significant immune escape as compared to that observed against the wild-type, BA.1 and BA.5 strains.^6^^,^^7^^,^^11^, ^12^, ^13^, ^14^, ^15^ However, neutralising assays for previous sub-lineages have often shown reduced neutralising which has not translated to a reduction in the real-world effectiveness against severe disease outcomes.^16^^,^^17^ To our knowledge, there are no real-world estimates of VE against BQ.1 or CH.1.1. Studies from the United States (US) found VE against infection and hospitalisation with XBB/XBB.1.5 was generally comparable to that seen against BA.5,^18^^,^^19^ while a study from Singapore found protection against A&E attendance was 49% during an XBB wave.^20^

As part of the UK COVID-19 vaccination programme, an autumn 2022 booster programme commencing 5th September 2022 was recommended by the JCVI and bivalent BA.1 boosters with either Pfizer BioNTech (Original/Omicron BA.1 Comirnaty®) or Moderna (Spikevax® bivalent Original/Omicron BA.1 vaccine) were offered to all adults aged 50 years and over and vulnerable individuals, including the immunosuppressed.^21^^,^^22^ Other countries, such as the United States, used bivalent BA.4-5 vaccines as part of their autumn 2022 booster programmes.^23^

Here, we use national-level electronic health records from England to estimate the incremental vaccine effectiveness (iVE), often also called relative VE,^23^^,^^24^ of the bivalent BA.1 boosters against hospitalisation with BQ.1, CH.1.1. and XBB.1.5 in England. We also assess the relative severity of these variants by estimating the odds of hospital admission or death following accident and emergency (A&E) attendance, for both CH.1.1 and XBB.1.5 compared to BQ.1. As a secondary indicator of variant severity, we estimate the odds of intensive care unit (ICU) admission or death amongst hospitalised cases by variant.

## Methods

### Study design

To estimate VE of the bivalent BA.1 booster vaccines offered as part of the autumn 2022 booster programme against hospitalisation by variant, a TNCC study design was used where positive PCR tests from hospitalised individuals aged 50 years and older are cases while negative tests from such individuals are controls, as previously described.^16^^,^^17^^,^^24^, ^25^, ^26^, ^27^

To estimate the odds of hospital admission by variant, individuals of any age with a positive PCR test attending A&E who went on to be admitted or transferred with a length of stay of 2 or more days, or whose attendance ended in death, or who died within 2 days of their A&E attendance were included, as well as comparable individuals who did not go on to be admitted, as previously described.^8^

To estimate the odds of ICU admission or death (referred to in this manuscript as severe outcomes following hospitalisation) amongst cases admitted to hospital, individuals aged 50 years and older who were hospitalised with COVID-19 and who were admitted to ICU or who died were included, as well as comparable individuals who did not require ICU and did not die. Additionally, a Cox proportional hazards survival regression was used to investigate length of stay amongst hospitalised cases by variant.

### Data sources

Full details of all data sources are available in the Supplementary Appendix. All data sources are national-level healthcare datasets which include the entire relevant population in England. The study period for tests contributing to all analyses was from 5th December 2022 to 2nd April 2023, when the variants of interest were co-circulating.

To estimate VE, hospital based positive and negative PCR tests were extracted, as previously described.^16^^,^^17^^,^^24^, ^25^, ^26^ To estimate the odds of hospital admission and severe outcomes in hospital, only PCR positive individuals were included. Variant status was identified by whole genome sequencing information from the national variant line list, coordinated by the COVID-19 Genomics UK consortium.^28^ Only individuals with BQ.1, CH.1.1 or XBB.1.5 were retained.

Data were linked to the National Immunisation Management System NIMS as previously described^16^^,^^17^^,^^24^, ^25^, ^26^^,^^29^ and accessed for dates of vaccination and manufacturer, sex, date of birth, date of death, ethnicity, and residential address. Data on health/social care worker status and risk group status (those identified as being at risk, clinically extremely vulnerable (CEV) or severely immunosuppressed previously in the pandemic and those identified recently as requiring an autumn booster due to a clinical risk factor by the NHS CaaS (Cohorting as a Service^30^)) and were also extracted from the NIMS.

For VE analyses the following individuals were excluded: those who were unvaccinated, those who had received only one dose, trial doses, an autumn dose without receiving at least two other doses prior to 5th September, an autumn booster less than 12 weeks after their next most recent dose, two autumn doses, a vaccine coded as bivalent prior to 5th September, an autumn dose not coded as bivalent, and those whose last dose prior to 5th September 2022 was by a manufacturer other than AstraZeneca, Pfizer or Moderna.^24^

To assess the odds of hospital admission following A&E attendance, cases were linked to the Emergency Care Data Set (ECDS) and Secondary Uses Service (SUS). Only those who attended A&E on the same day as their first positive test were included.^31^ SUS data were used to identify subsequent hospital admissions where the length of stay was at least 2 days. SUS data was also used to estimate VE against hospitalisation and the odds of ICU or death amongst those hospitalised, regardless of ECDS admission status. Admissions were restricted to those with a date of test 1 day before up to 2 days after the admission date and where the length of stay was at least 2 days. ICD-10 codes from the primary diagnosis field were used to classify acute respiratory illness (ARI) (Supplementary Table S1). Classification of Interventions and Procedures (OPCS-4) codes were used to identify individuals who received treatment on intensive care unit (ICU).^25^

### Covariates and adjustment

For all analyses, week of test date, gender, age group, region, IMD quintile and reinfection status were included as potential confounding variables. For VE and odds of ICU admission or death and length of stay analyses, ethnicity, risk group status, care home status and health and social care worker status were included as additional confounders. Vaccination status was an additional confounder for all severity analyses.

### Statistical analysis

To estimate VE, multivariable logistic regression was used with the test result as the outcome, vaccination status as the primary variable of interest and with confounder adjustment as described above. VE was calculated as 1- odds ratio and given as a percentage. Estimates were not shown where there were less than 30 controls or where the 95% CI lower bound was <−30% AND the top bound was >50% due to lack of precision. Incremental VE of the bivalent booster was estimated amongst those who had received at least two doses prior to the 5th September 2022 and whose final dose prior to the 5th September 2022 was at least 6 months before their test date, with those who did not receive a bivalent booster being the comparator group. VE was estimated at the following intervals since booster vaccination; 0–6 days, 7–13 days, 2–4 weeks, 5–9 weeks, 10–14 weeks, or 15 or more weeks. Analyses were restricted to those aged 50 years and older and estimated by manufacturers combined. Sensitivity analyses were conducted to estimate iVE for those with ARI ICD-10 coding in the primary diagnosis field.

To assess the odds of admission or death following A&E attendance by variant, conditional logistic regression models were used to estimate odds ratios of the outcome for both CH.1.1 and XBB.1.5 compared to BQ.1. Models were stratified by test week and with confounder adjustment as described above.

To estimate the odds of ICU or death amongst hospitalised cases by variant, multivariable logistic regression with ICU admission or death as the outcome, variant as the primary variable of interest and with confounder adjustment as described above. Sensitivity analyses were conducted to estimate the odds of ICU admission or death for those with ARI ICD-10 coding in the primary diagnosis field.

To estimate length of stay amongst hospitalised cases, we show the median length of stay and interquartile range (IQR). We also used a Cox proportional hazards survival regression where variant was included as an independent variable with confounder adjustment as described above. There was no significant deviation from the proportional hazards assumption of the Cox models. The predicted median length of stay is the point at the predicted 50% survival to discharge in the Cox model. Only individuals who had an admission date, discharge date and a length of stay between 0 and 21 days (to ensure all individuals in the study period had time to be discharged and to allow for delays in the SUS hospitalisation data reporting) were included. For some individuals with intervals of longer than 21 days we will not know about their length of stay being at least 21 days because they will not yet have been discharged at the time of the data extraction. These censored data are unobserved and cannot be analysed using censored data approaches so are excluded. The analysis was stratified by those who died and those who did not die to avoid bias in using this as a severity measure since some of the most severe cases will die after a short stay. Model outputs are reported as the predicted median length of stay.

### Role of the funding source

No external funding.

## Results

### Vaccine effectiveness against hospitalisation

**Fig. 1:**
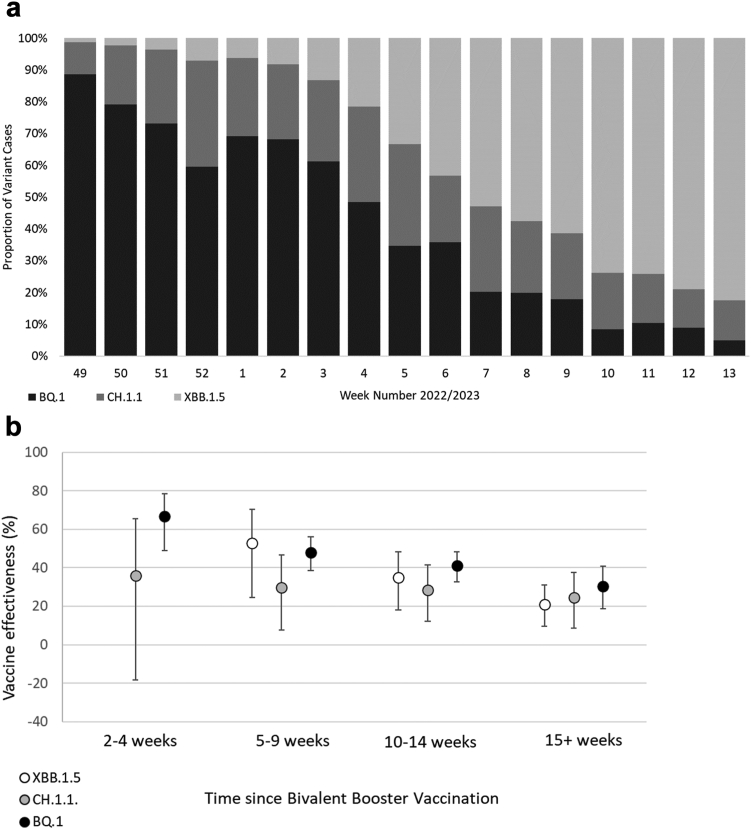
a) Proportion of BQ.1, CH.1.1, and XBB.1.5 variant cases over time in the study period. b) Incremental vaccine effectiveness (iVE) against hospitalisation of the bivalent BA.1 booster vaccine against BQ.1, CH.1.1 and XBB.1.5 amongst adults aged 50 years and older in England.

There were 191,229 eligible tests from hospitalised individuals aged 50 years and older, with 1647 BQ.1 cases, 877 CH.1.1 cases, 1357 XBB.1.5 cases and 187,348 test negative controls. Of all positive tests, 22% had sequencing information associated. The proportion of variant cases by week over time in shown in Fig. 1a and full descriptive characteristics of eligible tests are available in Supplementary Table S2.

The iVE of the bivalent BA.1 boosters was 48.0% (95% C.I.; 38.5–56.0%), 29.7% (95% C.I.; 7.5–46.6%) and 52.7% (95% C.I.; 24.6–70.4%), in addition to the protection from previous doses, against BQ.1, CH.1.1 and XBB.1.5, respectively, at 5–9 weeks post vaccination (Table 1, Fig. 1b). iVE against all sub-lineages waned over time, and iVE was 30.5% (95% C.I.; 18.7–40.6%), 24.5% (95% C.I.; 8.6–37.7%) and 21.1% (95% C.I.; 9.6–31.1%) against BQ.1, CH.1.1 and XBB.1.5, respectively, at 15 or more weeks post vaccination (Table 1, Fig. 1b). Point estimates were lower for CH.1.1 and XBB.1.5 than for BQ.1 at most time points, but confidence intervals overlapped and this difference was not statistically significant. Sensitivity analyses found there was no difference in iVE when we restricted to only include hospitalisations with a respiratory code in the primary diagnosis field or when we removed the adjustment for past positivity (Supplementary Table S3).

**Table 1:** Incremental vaccine effectiveness (iVE) against hospitalisation of the bivalent BA.1 booster vaccine against BQ.1, CH.1.1 and XBB.1.5 amongst adults aged 50 years and older in England.

Bivalent vaccine	Interval (weeks)	Controls	BQ.1		CH.1.1		XBB.1.5	
		n	n	VE (95% C.I.)	n	VE (95% C.I.)	n	VE (95% C.I.)
None	–	48,099	509	Baseline	211		338	
Pfizer or Moderna	2–4	3374	22	66.7 (48.7–78.4)	11	36 (−18.3 to 65.4)	6	n too small
	5–9	20,700	218	48.0 (38.5–56.0)	80	29.7 (7.5–46.6)	20	52.7 (24.6–70.4)
	10–14	45,087	495	41.1 (32.8–48.3)	214	28.3 (12.2–41.5)	109	35.0 (18.1–48.4)
	15+	69,263	394	30.5 (18.7–40.6)	348	24.5 (8.6–37.7)	883	21.1 (9.6–31.1)

### Odds of admission or death after A&E attendance

4665 individuals were identified who tested positive with BQ.1, CH.1.1 or XBB.1.5, and had a record of attendance to A&E on the same day as their positive test. Overall, the patient characteristics were broadly similar across the cohort (Supplementary Table S4). The majority of the cohort had received two or more vaccine doses, boosters, and the more recent autumn booster.

After adjusting by age group, sex, vaccination status, reinfection status, IMD quintile and geographical region, and stratifying by specimen test week, there was a significant reduction in odds after adjustment in CH.1.1 compared to BQ.1, although the upper confidence interval nears 1 (OR 0.87, 95% C.I.; 0.77–0.99; Table 2). There was no significant reduction in odds after adjustment in XBB.1.5, (OR 0.88, 95% C.I.; 0.75–1.02).

**Table 2:** Adjusted odds ratios (OR) and 95% confidence intervals (CI) comparing risk of admission or death among individuals who attended A&E with CH.1.1 and XBB.1.5 as compared to BQ.1.

	Total population	No hospital admission outcome	Hospital admission outcome	%	Adjusted Odds Ratio (95% CI)	P value
BQ.1	1724	862	862	50.0	Baseline	–
CH.1.1	1047	660	387	37.0	0.87 (0.77–0.99)	0.04
XBB.1.5	1894	1349	545	28.8	0.88 (0.75–1.02)	0.08

### Odds of admission to ICU or death after hospitalisation

Compared to the baseline of BQ.1, there was no significant difference in the odds of admission to ICU or death for those with XBB.1.5 (OR 0.67, 95% C.I.; 0.44–1.02) or CH.1.1 (OR 0.96, 95% C.I.; 0.71–1.30) compared to BQ.1 (Table 3). Sensitivity analyses restricting to individuals with a respiratory code in their primary diagnosis field found there was a significant reduction in the odds of admission to ICU or death for those with XBB.1.5 (OR 0.48, 95% C.I.; 0.25–0.89), but not with CH.1.1 (OR 0.79, 95% C.I.; 0.53–1.17) (Supplementary Fig. S2 and Table S5).

**Table 3:** Adjusted odds ratios (OR) and 95% confidence intervals (CI) comparing risk of ICU admission or death among individuals who were admitted to hospital, and had a length of stay of two or more days, with CH.1.1 and XBB.1.5 as compared to BQ.1.

	Total population	No ICU or death outcome	ICU or death outcome	%	Adjusted ORs (95% CI)	P value
BQ.1	1437	1235	202	14.1	Baseline	–
CH.1.1	613	530	83	13.5	0.96 (0.71–1.30)	0.80
XBB.1.5	561	506	55	9.8	0.67 (0.44–1.02)	0.06

### Median length of stay after hospital admission

The distribution of the lengths of hospital stays of cases by variant, stratified by individuals who went on to die and individuals who did not go on to die, is shown in Supplementary Fig. S3. The median lengths of stay were very similar by variant; both for individuals who died and individuals who did not go to die, and the length of stay was generally longer amongst individuals who went on to die (Table 4). The predicted medians were also estimated with adjustment for confounders; here there was no significant difference in the length of stay for those hospitalised with BQ.1 (predicted median length of stay 5.2 days; 95% C.I.; 4.9–5.6 days), CH.1.1 (predicted median length of stay 5.1 days; 95% C.I.; 4.6–5.6 days) or XBB.1.5 (predicted median length of stay 5.2 days; 95% C.I.; 4.6–5.8 days) (Table 4). Sensitivity analyses restricting to those with a respiratory code in their primary diagnosis field also found no significant difference in the length of stay by variant (Supplementary Table S6).

**Table 4:** Median length of stay with interquartile range and adjusted values of predicted median length of stay with 95% confidence intervals (CI) of individuals who were admitted to hospital with CH.1.1 and XBB.1.5 as compared to BQ.1, stratified by death status.

Variant	N	Median length of stay (IQR) (days)	Predicted median length of stay (95% CI) (days)^a^
Those who did not die			
BQ.1	1126	5 (2–11)	5.2 (4.9–5.6)
CH.1.1	518	5 (2–9)	5.1 (4.6–5.6)
XBB.1.5	518	6 (2–11)	5.2 (4.6–5.8)
Those who died			
BQ.1	282	8 (4–13)	6.4 (5.5–7.2)
CH.1.1	114	8 (3–13)	6.3 (5–7.6)
XBB.1.5	64	8.5 (4–13)	5.7 (3.8–7.6)

a Cox proportional hazards model with adjustment for week of test date, gender, age group, region, IMD quintile, reinfection status, ethnicity, risk group status, care home status, health and social care worker status and vaccination status.

## Discussion

We found no significant difference in the protection conferred by the bivalent BA.1 booster vaccines against hospitalisation with BQ.1, CH.1.1 and XBB.1.5. Point estimates for odds of severe disease indicators with CH.1.1 and XBB.1.5 were generally lower than for BQ.1, both for odds of hospital admission or death following A&E attendance; and for odds of ICU admission or death among hospitalised patients, though in most analyses this was not statistically significant. The length of stay following hospital admission also did not differ by variant.

These results follow previously observed trends, most recently with BA.4 and BA.5, showing no difference by sub-lineage in odds of admission or death following A&E attendance compared to the baseline of BA.2,^8^ and similarly with BA.4.6, BA.2.75 and BQ.1 compared to BA.5.^9^ Previously, we observed that VE against hospitalisation with BA.2, BA.4 and BA.5 peaked at around 60% at 2–14 weeks post vaccination following a third or fourth dose, estimated relative to those with waned immunity who had received their second dose at least 25 weeks prior.^17^ We here found the incremental effectiveness of the vaccines in addition to at least two doses of vaccine peaked at around 48.0%, 29.7% and 52.7% for BQ.1, CH.1.1 and XBB.1.5 5–9 week post-vaccination. Differences in testing policy, the vaccines given and the infection histories between the study periods make it difficult to directly compare estimates, but this could indicate effectiveness is slightly reduced for current circulating sub-lineages as compared to BA.2, BA.4 and BA.5.

Since most of the adult population in England has received multiple vaccine doses and very few individuals remain unvaccinated, we considered it most relevant to estimate the additional protection the booster gave on top of that which most of the adult population eligible for an autumn booster already had.^24^ A TNCC study from the US^18^ found a lower VE (around 40%) against infection with XBB/XBB.1.5 than that observed here, likely as VE is higher against more severe outcomes.^16^^,^^23^^,^^27^ A study from Singapore^20^ found similar VE against XBB; in their study the VE of an mRNA vaccine (in addition to that conferred by past doses) against A&E attendance was 49% during an XBB wave.

The iVE of the bivalent BA.1 boosters was comparable to that we and others have observed previously of BA.1 and BA.4-5 bivalent boosters, with evidence of waning at 15 or more weeks post-vaccination.^23^^,^^24^^,^^32^^,^^33^ Previously we have observed large difference in VE estimates when the hospitalisation outcome was not restricted to those with a respiratory code in the primary diagnosis field,^25^ however since September 2022, PCR testing in England has been restricted to those with respiratory disease in hospital settings and in our most recent analyses we have not observed a difference in VE.^24^ We therefore included all admissions regardless of ICD-10 coding in our primary VE analysis. Sensitivity analyses restricting to those with a respiratory code in the primary diagnosis field also showed no difference. We combined bivalent BA.1 booster manufacturers as we have previously not observed a difference between the Moderna BA.1 bivalent or Pfizer BA.1 bivalent vaccines.^24^

Our results do not indicate that there is a difference in the odds of an A&E presentation ending in hospital admission or death with CH.1.1 and XBB.1.5 as compared to BQ.1. Results for XBB.1.5 are consistent with previous evidence from the US on the proportion of individuals hospitalised^10^ and from Singapore in a community cohort showing no increased risk of hospitalisation.^34^ The end of freely available community testing for COVID-19 in England as well as the reduction in whole genome sequencing of positive tests has provided challenges in assessing the severity of emergent variants in England. Since tests performed in hospital settings were prioritised for sequencing, we have adapted our previous methodology^35^^,^^36^ to account for this sampling bias, a limitation noted in other studies,^34^ by restricting the analysis of relative severity just within individuals who attended A&E on the day of testing positive.^8^

We also found no difference in severity when assessing the odds of ICU admission or death, and by investigating the length of stay, amongst older adults. We considered the most severe outcomes were most relevant to investigate in older adults and restricted to those aged 50 and older. Sensitivity analyses restricting to those with a respiratory code in the primary diagnosis field found a decrease in the odds of ICU admission or death for XBB.1.5, as compared to BQ.1. No difference was found for CH.1.1, or for length of stay between any sub-lineage. It is possible that the decreased odds of ICU admission or death with a primary respiratory code with XBB.1.5 as compared to BQ.1 is a spurious finding due to smaller numbers of cases in the restricted analysis.

A strength of this study is the availability of real-time national-level surveillance data which has allowed us to rapidly investigate sub-lineages as they emerge. A key strength of the TNCC study design in estimating VE in contrast to a conventional cohort study or case-control design is that it helps to address unmeasured confounders related to differences in health seeking behaviours and infectious disease exposure between vaccinated and unvaccinated individuals. The TNCC requires testing to be independent of vaccination status, which is likely to be the case in a hospital setting with more severe cases. The methodology to assess the odds of hospital admission takes the approach of estimating relative severity just within cases who attended A&E. However, those attending A&E are more likely to experience severe infection than the general population. The analysis was restricted to those attending A&E on the same day as their first specimen date to account for this as those testing on the same day as presentation are more likely to represent the general population with limited access to free testing outside of healthcare settings.

Our study is an observational study that relies on hospital coding which can be prone to error. Similarly, given the observational nature of the study, there may be unmeasured confounders that we were unable to adjust for. Past infection may affect both VE and variant severity, however most infections are undocumented since freely available community testing ended. This missing data on past positivity may bias VE to be lower because past positivity is protective itself and associated with fewer vaccine doses. Not adjusting for known past positivity made little difference to estimates, as also shown in other studies,^24^ suggesting this is not likely to lead to a large bias. Sensitivity analyses (data not shown) demonstrated that inclusion of reinfection status had little effect on the estimates obtained on the odds of hospital admission following A&E attendance. Including those with past infection is most relevant to public health policy as most of the population have now been infected. A final limitation is that variant type was only available to a minority of the total positive tests from the study period.

Together, these results provide reassuring evidence that the bivalent BA.1 booster vaccines provide similar protection against hospitalisation with BQ.1, CH.1.1 and XBB.1.5, and that both the emergent CH.1.1 and XBB.1.5 sub-lineages do not cause more severe disease as compared to BQ.1. The analyses follow previously observed trends showing similarity in the vaccine effectiveness against, and severity of, Omicron sub-lineages.

## Contributors

JLB, NA, GD and MR conceptualised the study. NAA, FCMK, RJL, JS, KH, NAA developed the methodology. NG and MC provided the genomics data. FCMK, KH, RJL, RH and JS curated the data. FCMK, KH and RJL conducted the formal analysis, supported by NAA. FCMK, KH and RJL accessed and verified the data. FCMK and KH wrote the original draft of the manuscript. JLB, NA, MK, GD and MR provided supervision. All co-authors reviewed the manuscript and were responsible for the decision to submit the manuscript.

## Data sharing statement

This work is carried out under Regulation 3 of The Health Service (Control of Patient Information; Secretary of State for Health, 2002) using patient identification information without individual patient consent as part of the UKHSA legal requirement for public health surveillance and monitoring of vaccines. As such, authors cannot make the underlying dataset publicly available for ethical and legal reasons. However, all the data used for this analysis is included as aggregated data in the manuscript tables and appendix. Applications for relevant anonymised data should be submitted to the UKHSA Office for Data Release at https://www.gov.uk/government/publications/accessing-ukhsa-protected-data.

## Ethics committee approval

Surveillance of COVID-19 testing and vaccination is undertaken under Regulation 3 of The Health Service (Control of Patient Information) Regulations 2002 to collect confidential patient information (www.legislation.gov.uk/uksi/2002/1438/regulation/3/made) under Sections 3(i) (a) to (c), 3(i) (d) (i) and (ii) and 3 (3). The study protocol was subject to an internal review by the UK Health Security Agency Research Ethics and Governance Group and was found to be fully compliant with all regulatory requirements. As no regulatory issues were identified, and ethical review is not a requirement for this type of work, it was decided that a full ethical review would not be necessary.

## Declaration of interests

The Immunisation Department provides vaccine manufacturers (including Pfizer) with post-marketing surveillance reports about pneumococcal and meningococcal disease which the companies are required to submit to the UK Licensing authority in compliance with their Risk Management Strategy. A cost recovery charge is made for these reports. GD’s predecessor employer, Public Health England, received funding from GlaxoSmithKline for a research project related to seasonal influenza and antiviral treatment; this project preceded and had no relation to COVID-19, and GD had no role in and received no funding from the project. MK received consulting fees (Gilead Sciences Inc) for advising on development of a clinical module for collection patient-reported outcome data from people living with HIV. MK received honoraria (GESIDA (Spanish HIV/AIDS Association) for speaking at annual conference on patient-reported outcome measures for people with HIV.
